# *EWSR1-NFATC2* and *FUS-NFATC2* Gene Fusion-Associated Mesenchymal Tumors: Clinicopathologic Correlation and Literature Review

**DOI:** 10.1155/2019/9386390

**Published:** 2019-03-26

**Authors:** Beata Bode-Lesniewska, Christine Fritz, Gerhard Ulrich Exner, Ulrich Wagner, Bruno Fuchs

**Affiliations:** ^1^Institute of Pathology and Molecular Pathology, University Hospital, Zurich, Switzerland; ^2^Center of Orthopedics, Zurich, Switzerland; ^3^Department of Plastic and Reconstructive Surgery, University Hospital, Zurich, Switzerland; ^4^Department of Orthopedic Surgery, Cantonal Hospitals, Winterthur and Luzern, Switzerland

## Abstract

The spectrum of mesenchymal tumors associated with rearrangements of the *EWSR1* gene has been growing in recent years due to progress in molecular detection techniques. Originally identified as the gene involved in the pathogenesis of Ewing sarcoma, the *EWSR1* gene is now known to be rearranged in diverse clinical and histopathological entities. The *NFATC2* gene is one of the many translocation partners of *EWSR1* in gene fusions in a morphologically typical, albeit rare, subgroup of mesenchymal tumors. Little is known about the clinical characteristics of tumors containing *NFATC2* gene rearrangements since most of the few reports published describe molecular rather than clinical aspects. In the current study, we report three patients with tumors carrying the *EWSR1-NFATC2* gene translocation, including one rare primary tumor of soft tissues. Another patient with a benign-appearing bone tumor with a unique *FUS-NFATC2* gene translocation is described. In various mesenchymal tumors (e.g., myxoid/round cell liposarcoma, low-grade fibromyxoid sarcoma, or angiomatoid fibrous histiocytoma), the *FUS* gene, as a member of the TET family, may be alternatively rearranged instead of the *EWSR1* gene without any noticeable influence on the microscopical appearance or clinical outcome. This fact seems not to apply to mesenchymal tumors with the involvement of the *NFATC2* gene because both in our experience and according to the extensive literature review, they have different properties on the morphological and molecular level. Both *ESWSR1-NFATC2* and *FUS-NFATC2* fusion-carrying tumors do not show microscopical or clinical features of Ewing sarcoma.

## 1. Introduction

The current WHO classification [1] incorporates molecular alterations into the subtyping of mesenchymal soft tissue tumors, and the detection of specific genetic alteration is an important complement in standard histopathologic diagnostics [2, 3]. The *EWSR1* was the first gene identified in relation to the pathogenesis of a mesenchymal tumor in the early 90s [4]. Initially associated with the pathogenesis of Ewing sarcoma (ES), this gene is now known to play a pathogenic role in various mesenchymal and even some epithelial tumors [5–8]. *EWSR1* fusions with genes from the ETS transcription factor family [9–11] lead to the development of ES, a highly aggressive, undifferentiated, round cell tumor. In contrast, rearrangements of *EWSR1* with other partners are found in a morphological and clinical spectrum of entities, ranging from highly aggressive (clear cell sarcoma (CCS) and round cell-containing myxoid liposarcoma (RMLPS)) to less aggressive tumors (pure myxoid liposarcoma (MLPS) or extraskeletal myxoid chondrosarcoma (EMC)) [7, 8]. Some undifferentiated, round cell tumors without the classic *EWSR1* fusions to genes from the ETS family have been designated Ewing sarcoma-like tumors (ESLTs) [12–15]. Reproducible fusions *CIC-DUX4* [16] and *BCOR-CCNB3* [17] have been identified in subsets of the *EWSR1*-unrelated ESLT. Both the clinical course and therapy response seem to differ in such tumors compared to typical ES. For example, *CIC-DUX4* sarcomas are highly aggressive and do not respond to the ES chemotherapy [18], while *BCOR-CCNB3* sarcomas follow more indolent course [19].

Rare mesenchymal tumors carrying *EWSR1-NFATC2* fusions have been assigned to ESLTs, probably due to partial CD99 expression and/or involvement of the *EWSR1* [20–23]. However, recent studies demonstrate that not only histological features, but also the molecular profiles of such tumors differ from ESs [24–26]. Little is known about the malignant potential of mesenchymal tumors carrying rearrangements of the *NFATC2*, since even if reported and studied at the molecular level, the more detailed information of clinical course is given only for very few patients [12, 13, 20, 22–24, 26–30]. The histological appearance of *EWSR1-NFATC2* fusion-associated tumors is quite reproducible but shows microscopic heterogeneity and variability in the immunoprofile, not giving a pathognomonic pattern. There is no strict correlation of the *EWSR1-NFATC2* fusion tumors to any of the currently morphologically defined entity. Examples of these tumors have been identified among ESLTs: “myoepithelial tumors,” myoepithelioma-like “MHFs of bone,” or aggressive osteoblastomas (Table 1). The *EWSR1* FISH results with an amplification of the centromeric signal may serve as an important diagnostic hint.

Several mesenchymal tumors (e.g., ES, MLPS, or AFH) may contain fusions, in which *FUS* is alternatively rearranged instead of *EWSR1*. In such instances, no noticeable differences in morphology or clinical behaviour between *EWSR1*- and *FUS*-rearranged variants are observed. Few cases of *FUS-NFATC2* fusion-associated undifferentiated ESLTs have been reported in two recent studies [26, 33] with only limited clinicopathological information. The molecular profiles of *EWSR1-NFATC2* and *FUS-NFATC2* were, however, strikingly different [26].

In the current study, we describe three patients with *EWSR1-NFATC2* fusion-associated tumors. Initial histologic diagnoses were sclerosing epithelioid fibrosarcoma (SEF), myoepithelial tumor, and EMC-like tumor. We provide detailed histopathologic, immunohistochemical, molecular, and clinical information as a basis for better characterisation of this molecular category, emphasizing their distinction from ESs and ESLTs. An additional exceptional case of a clinically indolent and microscopically bland bone tumor mimicking aneurysmal bone cyst (ABC) containing the *FUS-NFATC2* fusion is described. *EWSR1-NFATC2-* and *FUS-NFATC2-*associated tumors are discussed in the context of the published literature.

## 2. Materials and Methods

Cases with detectable rearrangements of the *NFATC2* were retrieved from the files of the Institute of Pathology, University Hospital, Zurich, Switzerland. Two older cases (Cases 1 and 2) showing suggestive microscopical pattern and *EWSR1* FISH findings were studied by NGS retrospectively, while the other two cases are current and were studied by NGS included in the routine diagnostic work-up. Clinical and follow-up data were obtained from clinical databases of the involved institutions. The study was approved by Institutional Review Board (Cantonal Ethics Committee; KEK_ZH_2013_0430).

### 2.1. Histology and Immunohistochemistry

Tumor tissue samples were fixed in buffered 4% formalin, embedded in paraffin, and stained according to standard procedures. Immunohistochemistry (IHC) using the broad-spectrum cytokeratin (AE1/AE3, Dako, Baar, Switzerland), CD99 (12E7, Abcam Ltd., Cambridge, UK), EMA (E29, Dako AG, Baar, Switzerland), and MIB1 (30-9, Ventana Roche, Basel, Switzerland) antibodies was performed, using the Ventana Benchmark XT Automated system (Ventana Medical Systems, Tucson, Arizona).

### 2.2. Fluorescence In Situ Hybridization (FISH)

FISH studies were performed on formalin-fixed, paraffin-embedded, 2 *μ*m thick sections. Dual-color break-apart FISH detecting translocations involving the *EWSR1* and *FUS* genes (both from Vysis, Abbott AG, Baar, Switzerland) and *NR4A3* gene (ZytoVision GmbH, Bremerhaven, Germany) was performed using commercially available probes. Fluorescence staining was visualized with an Olympus BX61-microscope (Olympus, Volketswil, Switzerland) equipped with DAPI, SpectrumGreen, and SpectrumOrange filters. Images were acquired with a CCD camera and processed with AnalySIS software (Soft Imagining System, Munster, Germany). At least 50 nonoverlapping nuclei were analysed. If the sample contained at least 25% split red and green signals (separation by at least twice the distance occupied by a single probe), the tumor was regarded as rearranged.

### 2.3. Next Generation Sequencing (NGS)

High-throughput analysis was performed as previously described [34]. Briefly, RNA was extracted using the Maxwell 16 LEV RNA FFPE Purification Kit (Promega Corporation). Libraries were prepared using Anchored-Multiplex-PCR with the commercially available Archer FusionPlex Sarcoma Panel (ArcherDx, Boulder, CO). The RNA input was 250 ng, and cDNA was synthesized using random primers. Libraries were quantified using qPCR (KAPA Biosystems, South San Francisco, CA, USA). Samples were sequenced on the MiSeq platform (Illumina, San Diego, CA, USA). The resulting FASTQ files were analyzed using the standard RNA-fusion workflow as implemented in the Archer Analysis Suite 5.1.3.

## 3. Results

The clinical characteristics of the four patients are summarized in Table 2.

### 3.1. Histopathology and Immunohistochemistry

#### 3.1.1. Case 1

**Figure 1:**
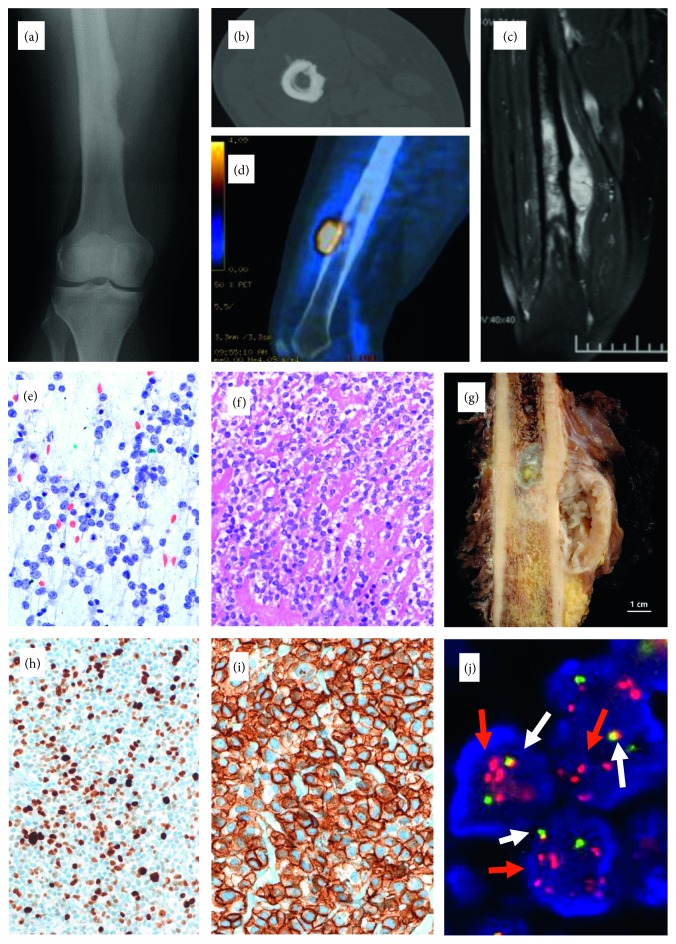
Tumor of the diaphysis of the right femur in a 34-year-old woman. Imaging studies ((a) conventional radiogram, (b) CT, (c) MRI, and (d) PET/CT) revealed a cortex-based mass with intraosseous and extraosseous extension, corresponding to the multilobulated tumor in the cut section of the resection specimen (g). (e) Direct smear of the biopsy (Papanikolaou stain, 400x) showed small, blue, round cell population. (f) On histologic examination, the tumor displayed a prominent collagen-rich extracellular matrix (H&E; 200x) with a moderate proliferation rate ((h) MIB1 immunohistochemistry; 100x) and strong CD99 expression ((i) 400). (j) Break-apart probe FISH analysis of the *EWSR1* gene showed one to two fused signals (white arrows) and low-grade amplified red signals (red arrows) in the tumor cell nuclei, suggesting the rearrangement of the *EWSR1* gene with additional chromosomal aberrations.

Core biopsy from a large intraosseous and extraosseous, cortex-based tumor of the diaphysis of the right femur in a 34-year-old woman (Figure 1) showed a partially necrotic, cellular proliferation of monomorphic, small, blue, and round cell population, which was embedded in a sclerotic stroma, resulting in a striking trabecular appearance. There was no evidence of typical osteoid or mineralisation. The immunophenotype was nonspecific (CK−, S100−, Des−, and CD45−); however diffuse CD99 expression was found. An unusual rearrangement pattern with an amplified and split red signal was found in the *EWSR1* FISH, which at the time of the diagnosis 11 years ago had not been previously described and was considered difficult to interpret and unusual for ES. The RT-PCR of the *EWSR1-FLI1* and *EWSR1-ERG* fusions was negative. Although largely a diagnosis of exclusion, the microscopic findings were considered consistent with sclerosing epithelioid fibrosarcoma (SEF). MUC4 immunostaining was not available at the time of the diagnosis and was negative retrospectively. No further tumor manifestations were found on staging. The clinical decision was made to treat the patient with preoperative chemotherapy according to the EURAMOS protocol [35]. Subsequent complete tumor resection revealed no chemotherapy-induced necrosis. 4.5 years after the initial presentation, a 1 cm skin metastasis on the ipsilateral thigh was completely excised and showed identical microscopical pattern as the primary. The patient was regularly followed, and 10.5 years after the first presentation, a solitary 7 mm lung metastasis was resected. Currently (11 years follow-up), the patient is doing well without known tumor manifestation. NGS analyses performed retrospectively on the skin metastasis revealed the *EWSR1-NFATC2* gene fusion.

#### 3.1.2. Case 2

**Figure 2:**
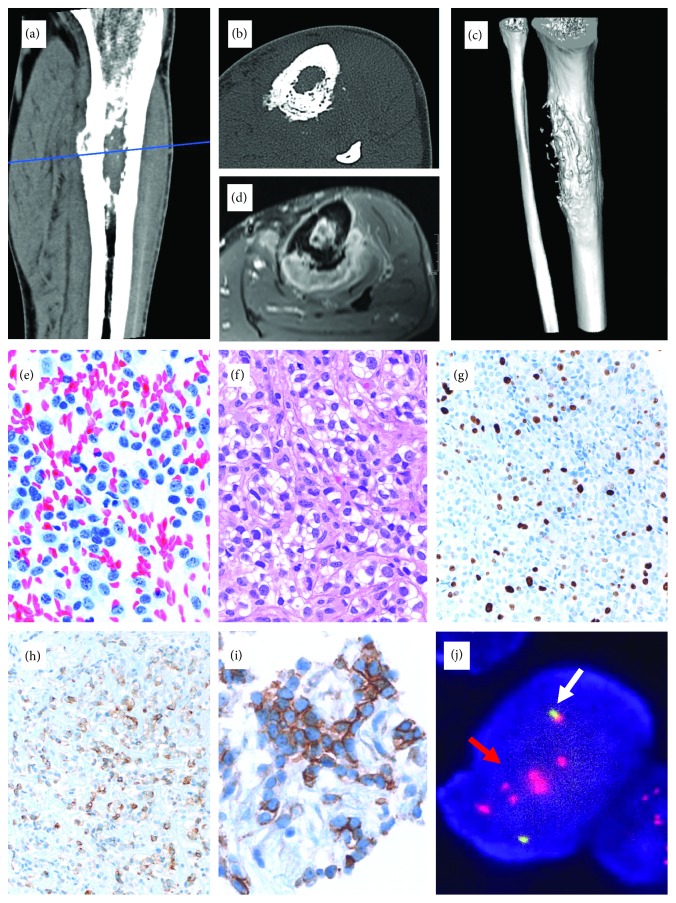
Imaging of a painful lesion of the proximal diaphysis of the left tibia in a 42-year-old man ((a, b) CT, (c) 3D CT reconstruction, and (d) MRI) showed a cortex-based mass with intraosseous and extraosseous extension. (e) Direct smear of the biopsy (Papanikolaou stain, 400x) showed a small, blue, round cell population. (f) Histological examination revealed tumor cells with clear cytoplasm, embedded in collagen-rich extracellular matrix (H&E; 200x). The cells have a low proliferation rate ((g) MIB1 immunohistochemistry; 100x) and focal cytokeratin (AE1/AE3) ((h) 200x) and CD99 expression ((i) 400). (j) On break-apart probe FISH analysis of the *EWSR1* gene, one to two fused signals (white arrows) and low-grade amplified red signals (red arrows) in the tumor cell nuclei were seen, suggesting rearrangement of the *EWSR1* gene with additional chromosomal aberrations.

Due to difficult histologic interpretation and extensive necrosis, two core biopsies were performed at external institutions, followed by the curettage of an intraosseous and extraosseous process of a 42-year-old man with clinical suspicion of an osteomyelitis of the left tibia (Figure 2). The tumor cells were small and inconspicuous, grouped in solid nests and trabecula, embedded in collagen-rich matrix. The cytoplasm was clear with distinct borders. There were few mitoses and a low proliferation index. The immunophenotype revealed diffuse, weak expression of cytokeratin, focal CD99 positivity, and coexpression of EMA, CD10, and CD117. Immunolabeling for S100, SMA, desmin, p63, CD34, and CD45 was negative. Nuclear expression of INI1 was retained. *EWSR1* FISH showed a rearrangement pattern of an amplified split red signal. RT-PCRs of the *EWSR1-FLI1* and *EWSR1-ERG* fusions were negative. Based on the microscopic appearance, cytokeratin expression and low proliferation rate, the diagnosis of a primary low-grade myoepithelial carcinoma was rendered. No further tumor manifestations were found upon staging. Local complete resection with reconstruction was performed. Persistent surgical complications led to amputation 7 years later. No adjuvant therapy was given. Neither local nor systemic tumor manifestations were found with a follow-up of altogether 8.5 years. Retrospective NGS of the curettage sample revealed the *EWSR1-NFATC2* fusion.

#### 3.1.3. Case 3

**Figure 3:**
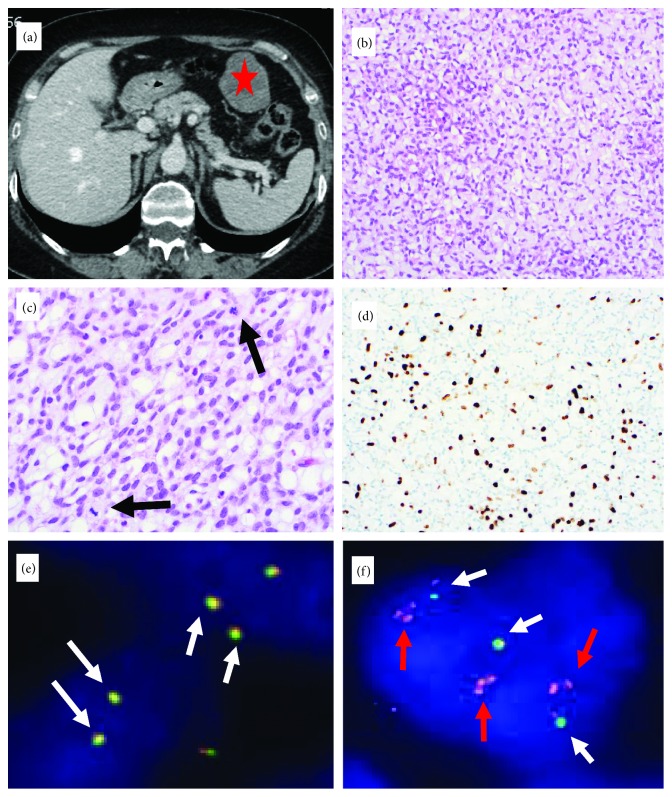
CT imaging in a 60-year-old woman showed an isolated upper intraabdominal mass ((a) red asterisk). (b) Moderately cellular tumor with a prominent chondromyxoid matrix and trabecular growth pattern (H&E stain; original magnification 200x). (c) Rare mitoses are present (arrows) (H&E; 400x). (d) Heterogeneous proliferation index of up to 30% (MIB1 immunohistochemistry; 200x). (e) No rearrangement of the *NR4A3* gene in FISH analysis, excluding extraskeletal myxoid chondrosarcoma (arrows: exclusively fused signals). (f) Break-apart FISH analysis of the *EWSR1* gene demonstrated an unusual pattern of one to three fused signals (white arrows) and low-grade amplified red signals (red arrows) in tumor cell nuclei, suggesting rearrangement of the *EWSR1* gene with additional chromosomal aberrations.

Core biopsy of an intra-abdominal epigastric mass in a 60-year-old woman (Figure 3) showed mesenchymal tumor with abundant extracellular matrix, composed of a trabecular network of monomorphic cells. Immunohistochemistry was negative for GIST markers as well as S100, HMB45, SMA, desmin, synaptophysin, STAT6, and MUC4. The nuclear expression of INI1 was retained. Rare cells expressed cytokeratin and EMA. Diffuse weak CD99 expression was observed. The differential diagnosis included EMC and myoepithelial tumor. As both categories may show rearrangement of the *EWSR1*, FISH was performed revealing rearrangement with low-level amplification of the red signal. *NR4A3* FISH showed a normal pattern, excluding the diagnosis of EMC. NGS analysis of the resection specimen revealed the *EWSR1-NFATC2* fusion. No adjuvant therapy was given, and no further tumor manifestations were found upon staging.

#### 3.1.4. Case 4

**Figure 4:**
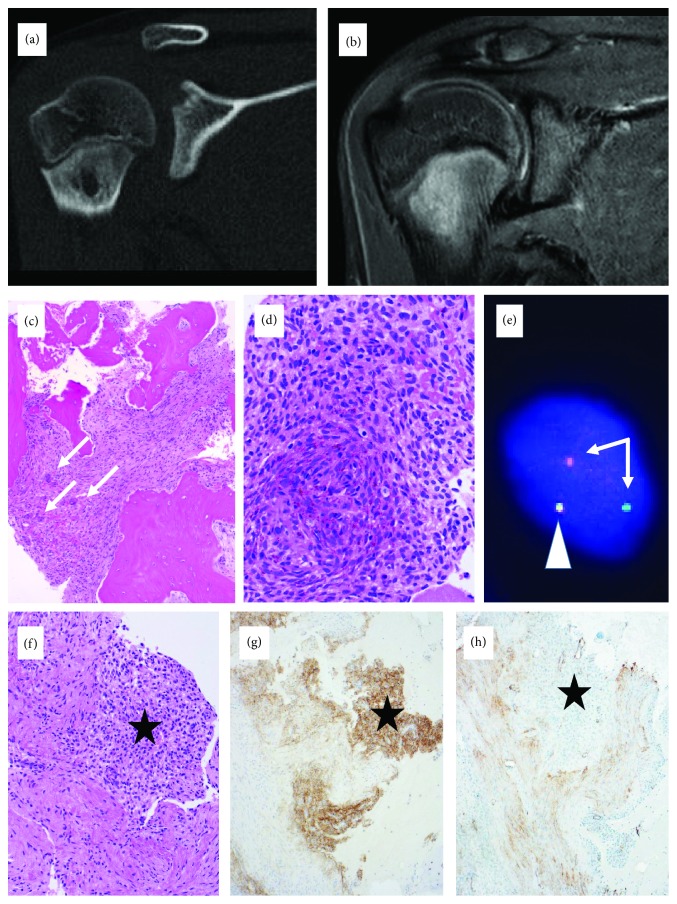
Symptomatic bone lesion in the metaphysis of the right proximal humerus of a 12-year-old boy. (a) CT and (b) MRI imaging demonstrated an irregular, partially osteolytic lesion. (c) Core biopsy revealed giant cells (arrows) containing bland spindle cell proliferation. (d) Curettage showed similar lesional tissue without necrosis, pleomorphism, or mitotic activity; however, a conspicuous biphasic pattern was observed (d, f). The distinctive spindle cell population contained siderin and mast cells and expressed immunohistochemically EMA (g) and CD99 (not shown), while the adjacent EMA and CD99 negative areas showed SMA expression (h). (e) FISH analysis with a break-apart probe demonstrated one fused (arrowhead) and one split (arrows) signal, indicating the rearrangement of the *FUS* gene.

Core biopsy of a metaphyseal mass of the right humerus in a 12-year-old boy (Figure 4) revealed an intramedullar bland spindle-cell proliferation with focal siderin depositions and few osteoclast-type giant cells. No necrosis or mitotic activity was observed. There was no osteoid or cartilage production. Given the radiologic differential diagnosis of an aneurysmal bone cyst (ABC), *USP6* FISH was performed which was inconclusive. NGS, initially performed on the core biopsy and repeated independently on the subsequent curettage, revealed the same *FUS-NFATC2* fusion in both specimens. *FUS* FISH performed for verification of the NGS showed the classical break-apart pattern without amplification. The lesional tissue showed very low proliferative activity in the MIB1 staining (<5%). Diffuse expression of EMA and CD99 was seen, while the reaction for SMA, desmin, S100, CD34, and synaptophysin remained negative. The curettage did not contain high-grade tumor. At 8 months follow-up, there was no evidence of recurrent tumor on the control MRI.

### 3.2. Fluorescence In Situ Hybridization (FISH)/Next Generation Sequencing (NGS)

In cases 1, 2, and 3, the break-apart probe for the *EWSR1* gene (Figures 1(j), 2(j), and 3(f)) showed an unusual pattern for break-apart probes with one to three fused signals and several grouped and amplified red signals. This pattern diagnostic of gene rearrangement, however, indicates that the break within chromosome 22q is associated with additional aberrations. Since a differential diagnosis of EMC was considered in case 3, a second *NR4A3* FISH reaction was performed and was negative (Figure 3(e)). In all three cases 1, 2, and 3, an *EWSR1-NFATC2* fusion was found on NGS.

The differential diagnosis in case 4 of the osteolytic (Figures 4(a) and 4(b)), giant cell containing histologically bland proliferation (Figures 4(c)–4(f)) in the metaphysis of a young patient included an ABC. *USP6* FISH was inconclusive, suggesting the lack of the rearrangement. A *FUS-NFATC2* gene fusion was detected on NGS performed independently and metachronically, both on the core biopsy as well as the curettage specimen. This result was verified with *FUS* FISH, which showed classical break-apart pattern with one fused and one split signal in both samples (Figure 4(e)).

### 3.3. Follow-Up

All four patients are currently alive and without tumor manifestations. Patient 1 developed skin metastasis involving the thigh, four and a half years following the first diagnosis and a small lung metastasis six years later. Both lesions were completely resected without additional therapy. Patient 2 suffered several local surgical complications, which resulted in amputation of the lower leg at 7.5 years after the initial diagnosis. At 8.5 years after the diagnosis, the patient is free of tumor at 8.5 years without adjuvant therapy. The 8-month follow-up for patients 3 and 4 is still rather short to draw final conclusions; however, both patients are free of tumor.

## 4. Review of the Published Literature

### 4.1. *EWSR1-NFATC2*

Review of the published literature in the PubMed databank revealed 8 papers with at least minimal clinical information on 15 patients with tumors carrying the *EWSR1-NFATC2* fusion [12, 13, 22, 26–30] (Table 1). Fourteen more cases lacking clinical information are mentioned in three further studies with a special focus on the molecular profiling of ES and ESLT [20, 23, 24]. Of the cases with clinical information, there was a striking male predominance with 14 males and 4 females. Of the 17 patients with adequate data, only three where younger than 18; the youngest patient reported was 12-year-old and the oldest 61.5-year-old (median 30, mean age 31.5 years). Only three cases were described in soft tissue (two in the calf and one intra-abdominal), while the rest occurred in long bones (8 femur, 3 humerus, 2 tibia, 1 fibula; one tumor “lower limb”). Follow-up data are available for 9 patients (including 3 patients of the current study) with all reported as alive, with follow-up ranging from 8 months to 11 years (mean 52 months). In case of the tenth patient, who is reportedly alive and tumor-free, no follow-up period is specified. Metastases have been detected in only two patients (one with lung and bone metastases and the other with skin and lung metastases) and local recurrence in 3 patients. The data on treatment are even more scarce: six patients underwent chemotherapy (including ES protocol in 4 patients, one for falsely diagnosed lymphoma, and one EURAMOS) and were treated by surgery.

### 4.2. *FUS-NFATC2*

Two publications describe 4 patients harbouring tumors the *FUS-NFATC2* fusion detected in the context of molecular profiling of ES/ESLT [26, 33] (Table 3). Gender and age (three male patients: 15-, 22-, and 43-year-olds and a 49-year-old woman) as well as tumor locations (all in femur) are given; however, no details on therapy or especially follow-up or outcome are provided. Histologically, they are described as mitotically active with necrosis and areas comprising both round cell and spindle cell features, accompanied by focal chondroid differentiation or myxohyaline stroma [26, 33]. Molecular profiling with clustering analyses [26] suggests that there is a substantial difference between the *FUS-NFATC2* and *EWSR1-NFATC2* tumors since they show distinct and unrelated signatures.

The patient described in the current study is the youngest reported with a tumor harbouring *FUS-NFATC2* fusion. In addition, this tumor represents the first case with a *FUS-NFATC2* fusion without microscopic or clinical evidence of high-grade malignancy.

## 5. Discussion

Following the recognition that Ewing sarcoma is pathogenically caused by translocations of a gene located on chromosome 22q12 (subsequently called Ewing sarcoma gene) to partner genes of the ETS family of transforming factors, a subgroup of mostly aggressive sarcomas not carrying this translocation type has been identified and designed as “Ewing sarcoma-like tumors” (ESLT) [12–15]. The term ESLT has subsequently been applied to some unclassifiable tumors, which did not fit in any category of established entities and while only remotely resembling ES, showed variable immunohistochemical expression of CD99 antigen and/or the involvement of *EWSR1*, not linked to a partner from the ETS family. *EWSR1* rearrangements are involved in the pathogenesis of several well-established clinically and morphologically obviously non-ES, non-ESLT, entities such as MLPS, CCS, AFH, and a subgroup of myoepithelial tumors of soft tissue [7, 8].

In the current study, we describe three patients with tumors characterised by the *EWSR1-NFATC2* fusion. *NFATC2* (nuclear factor of activated T-cells, calcineurin-dependent 2) gene is involved in the function of the activated T-cell transcription complex [21]. Several studies (Table 1) describe tumors carrying *EWSR1-NFATC2* fusion, mostly identified among tumors at first classified as ESLTs. The comparison of the histopathology of these tumors suggests that they show a quite typical microscopic appearance with trabecular growth pattern and sclerotic, myxohyaline matrix, which substantially differs from the round cell aspect of typically stroma-devoid ESs and in fact resembles rather neoplasias from the spectrum of myoepithelial tumors. Rearrangements of *EWSR1* with various partners [36–40] have been described in a subgroup of soft tissue myoepithelial tumors. Other subgroups of myoepithelial tumors of soft tissue are associated with alternative genetic aberrations such as rearrangements of other genes (e.g., *PLAG1* [41]) or homozygous deletion of the INI1/*SMARCB1* gene [42–44], while in a large proportion, the genetic background is not yet known. In fact, one of the tumors of the current series was initially diagnosed as a myoepithelial carcinoma due to the expression of cytokeratins and the presence of the *EWSR1* rearrangement. The intra-abdominal tumor of patient 3 was initially considered to be either a myoepithelial tumor or an EMC based on the rearrangement of the *EWSR1*. Another tumor reported in the literature was identified as myoepithelioma-like sarcoma of bone [29] in retrospective work-up of 57 undifferentiated bone sarcomas.

The first case of our series with currently the longest reported follow-up in the literature was diagnosed 11 years ago as a sclerosing epithelioid fibrosarcoma (SEF), which already then was considered to be an exclusion diagnosis. At the time of the initial diagnosis, the concept of primary myoepithelial tumors of soft tissue and bones was not yet well established and little was known on the molecular background of SEF and the MUC4 immunohistochemistry—nowadays easy to apply to verify this diagnosis—was not yet available.

The molecular hallmark of the *EWSR1-NFATC2* fusion is the accompanying secondary structural aberration of the fusion product, which leads to low-level amplification of the centromeric portion of the probe on the break-apart FISH. This pattern is very typical and was observed in all cases reported so far. In fact, this finding should serve as a first diagnostic hint of the *EWSR1-NFATC2*-translocated sarcomas.

Tumors carrying the *EWSR1-NFATC2* fusion have been reported mostly in long bones of male patients with only few reports in soft tissue or in female patients. The intra-abdominal tumor of the female patient 3 of our series is to the best of our knowledge the first reported in a nonextremity location. Patients with *EWSR1-NFATC2* tumors are generally older than ES patients, with the median age of 30 years at presentation for the cases with reported age data, even if these tumors do occur below the age of 20. Apart from local destructive growth, a fraction of reported patients developed lung and rare skin or bone metastases, confirming the malignant potential of *EWSR1-NFATC2* fusion-associated tumors. However, long survival periods, even without adjuvant therapy and the lack of reported deaths due to tumor, indicate that these tumors belong to the low-grade malignant category. The relation of *EWSR1-NFATC2*-translocated tumors to the group of ESs and ESLTs is currently being controversially discussed in the literature [27, 45, 46]. Based on our experience and the review of the data from the published literature, these tumors merit consideration of a separate category.

The *FUS* gene as a member of the TET/FET family has been found to be alternatively rearranged instead of *EWSR1* in several mesenchymal entities such as ES, MLPS, LGFMS, or AFH, without noticeable differences in morphology or clinical course. The *FUS* similar to *EWSR1* codes for a TET family member of RNA-binding proteins sharing homologous sentences. The 5′ transactivation domain of *FUS* and *EWSR1* seems to be interchangeable in terms of transforming potential, and the fusion proteins resulting from *EWSR1* or *FUS* rearrangements with any given partner seem to exert identical biological effects [14]. Interestingly, however, in cases of *FUS* gene-related ES, the fusion partners involve the ETS genes *ERG* or *FEV*, but not *FLI1*, which is the most commonly rearranged partner of *EWSR1* in ES. In two recent studies, isolated cases of *FUS-NFATC2* fusion associated tumors have been cited in the literature among undifferentiated ESLTs (Table 3) [26, 33] with only limited information on the histopathology and clinical features. Of note, the molecular profiles of *EWSR1-NFATC2* and *FUS-NFATC2* tumors were strikingly different. This observation suggests that contrary to previously described tumors with *EWSR1* and/or *FUS* genes fused to other genes than *NFATC2,* substantial differences must exist. The FISH pattern of the *FUS* rearrangement is not described in the two papers reporting the *FUS-NFATC2*-associated tumors. In case 4 of the current series, in contrast to the *EWSR1-NFATC2* fusion, the *FUS-NFATC2* fusion was not associated with amplification of the affected *FUS* region on FISH, implying a different structure of the fusion product. The histological features of case 4, which lack morphological and clinical evidence of malignancy, appear to differ from the three cases of ESLT described in the literature. More data are required regarding the clinical, histopathological, and molecular properties of tumors carrying the *FUS-NFATC2* fusion.

The current study and literature review underscore the observation that the *EWSR1-NFATC2* fusion-associated tumors are distinct histopathological and molecular entities that probably should not be included in the ESLT category. Only a handful of reported cases with incomplete clinical data are available on *FUS-NFATC2* fusion-associated neoplasia. However, interestingly, based on case 4 of the current study and previous molecular profiling data [26], there seems to be a difference between tumors having either the *ESWR1* or *FUS* as the fusion partner of *NFATC2* gene. As we move towards a molecular classification of mesenchymal tumors, much remains to be learned about the correlation of the specific genetic aberrations with the clinical outcome and the response to therapy.

## Figures and Tables

**Table 1:** Published data of patients with tumors containing EWSR1-NFATC2 fusion.

Literature case nr	Reference	Age (y)	Gender	Site	Original diagnosis	Follow-up/outcome	Therapy
1	[22, 31]	39	M	Humerus R	ESLT	15 mo after surgery lung met	Resection post-op Chth EuroEwing 99
						26 mo after surgery bone met spine	
						40 mo after surgery 2nd lung met	
						48 mo after surgery ANED	
2		16	M	Femur R	ESLT	ND	ND
3		21	M	Thigh R	ESLT	ND	ND
4		25	M	Femur R	ESLT	ND	ND
5	[29]	32	M	Lower limb	Myoepithelial-like “MFH” of bone 7 y before radiation due to lymphoma	64 mo; ANED (never metastasized)	ND
6	[28]	12	M	Femur	ES	11 mo; ANED (never metastasized)	Pre-op Chth resection
7		28	M	Humerus	Lymphoma	4 y local recurrence 4 y lung metastasis “possible”	Chth (“standard protocol”)
8	[12]	42	M	Femur R		ND	ND
9	[30]	30	M	Femur L	Aggressive osteoblastoma osteosarcoma (recurrence)	2.5 y post-op local rec 3.5 y ANED	Curettage and bone graft 2.5 y post-op Chth after local recurrence
10	[27]	24	F	Calf subcutis	Extraskeletal ES	12 mo; ANED (never recurrence or metastases)	Pre-op Chth Ewing protocol
11	[32]	ND	ND	Fibula	ESLT	ANED (how long?)	ND
12	[26]	32.7	M	Humerus	ES	ND	ND
13		12.7	F	Tibia	ES	ND	ND
14		61.5	M	Calf	ES	ND	ND
15		23.6	M	Femur	ES	ND	ND
16	Current study	34	F	Femur R	SEF	4.5 y skin metastasis 10.5 y lung metastasis 11 y ANED	Pre-op Chth EURAMOS protocol resection
17		42	M	Tibia L	Myoepithelal tumor	8.5 y ANED	Curettage resection amputation (due to surgical complications)
18		60	F	Abdomen	Myoepithelal tumor or EMC	8 mo ANED	Resection
19–24	[20]	ND	ND	ND	ES/ESLT	ND	ND
25	[23]	ND	ND	ND	ES/ESLT	ND	ND
26–32	[24]	ND	ND	ND	ES/ESLT	ND	ND

ANED: alive, no evidence of disease; Chth: chemotherapy; EMC: extraskeletal myxoid chondrosarcoma; ES: Ewing sarcoma; ESLT: Ewing sarcoma-like tumor; F: female; L: left; ND: no data; M: male; mo: months; R: right; SEF: sclerosing epithelioid fibrosarcoma; y: years.

**Table 2:** Clinical data of the study patients.

No.	Gender	Age (y)	Site	Primary Dg	Therapy	Follow-up	Outcome
1	F	34	Femur R	SEF	Neoadj. ChTh; resection	4.5 y skin met (thigh R); 8.5 y-lung met (OL R)	11 y; NED
2	M	42	Tibia L	Myoepithelial tumor	Curretage, resection 1 y later; amputation 7.5 y post-op due to surgical complications		8.5 y; NED
3	F	60	Upper abdomen	Myoepithelial tumor or EMC	Resection		8 mo; NED
4	M	12	Humerus R	Compatible with ABC	Intralesional curettage		8 mo; NED

ABC: aneurysmal bone cyst; ChTh: chemotherapy; EMC: extraskeletal myxoid chondrosarcoma; L: left; NED: no evidence of disease; R: right; SEF: sclerosing epithelioid fibrosarcoma; y: years.

**Table 3:** Published data of patients with tumors containing *FUS-NFATC2* fusion.

Literature case no.	Reference	Age (y)	Gender	Site	Original diagnosis	Follow-up/outcome	Therapy
1	[33]	15	M	Femur R	ES	ND	ND
2	[26]	33.3	M	Femur	Osteosarcoma	ND	ND
3		49	F	Femur	Unclassified small round cell sarcoma	ND	ND
4		43.3	M	Femur	ES or ESLT	ND	ND
5	Current study	12	M	Humerus R	ABC	8 mo ANED	Curettage

ABC: aneurysmal bone cyst; ANED: alive, no evidence of disease; ES: Ewing sarcoma; ESLT: Ewing sarcoma-like tumor; F: female; L: left; ND: no data; M: male; mo: months; R: right; y: years.

## Data Availability

The histopathological, immunohistochemical, and molecular data used to support the findings of this study are included within the article.
